# Introduction to the theme issue ‘Species' ranges in the face of changing environments’

**DOI:** 10.1098/rstb.2021.0002

**Published:** 2022-04-11

**Authors:** Marina Rafajlović, Jake M. Alexander, Roger K. Butlin, Kerstin Johannesson

**Affiliations:** ^1^ Department of Marine Sciences, University of Gothenburg, 405 30 Gothenburg, Sweden; ^2^ Centre for Marine Evolutionary Biology, University of Gothenburg, 405 30 Gothenburg, Sweden; ^3^ Department of Environmental Systems Science, ETH Zürich, 8092 Zürich, Switzerland; ^4^ Ecology and Evolutionary Biology, School of Biosciences, University of Sheffield, Sheffield S10 2TN, UK; ^5^ Department of Marine Sciences, University of Gothenburg, Tjärnö Marine Laboratory, 452 96 Strömstad, Sweden

**Keywords:** limits to species’ ranges, local adaptation, climate change evolution, range shift, range expansion and contraction, range fragmentation

## Abstract

Understanding where, when and how species’ ranges will be modified is both a fundamental problem and essential to predicting how spatio-temporal environmental changes in abiotic and biotic factors impact biodiversity. Notably, different species may respond disparately to similar environmental changes: some species may overcome an environmental change only with difficulty or not at all, while other species may readily overcome the same change. Ranges may contract, expand or move. The drivers and consequences of this variability in species' responses remain puzzling. Importantly, changes in a species’ range creates feedbacks to the environmental conditions, populations and communities in its previous and current range, rendering population genetic, population dynamic and community processes inextricably linked. Understanding these links is critical in guiding biodiversity management and conservation efforts. This theme issue presents current thinking about the factors and mechanisms that limit and/or modify species' ranges. It also outlines different approaches to detect changes in species’ distributions, and illustrates cases of range modifications in several taxa. Overall, this theme issue highlights the urgency of understanding species' ranges but shows that we are only just beginning to disentangle the processes involved. One way forward is to unite ecology with evolutionary biology and empirical with modelling approaches.

This article is part of the theme issue ‘Species’ ranges in the face of changing environments (Part II)’.

## 1. Introduction

Species’ ranges are temporally and spatially dynamic, undergoing expansions, contractions, shifts and/or local (de)fragmentation over time [1–7]. While range changes are mostly triggered by modifications in environmental factors, they can be underlain by ecological responses, evolutionary changes within species, or both. A change in one species' range may have impacts that extend to other species, and to the communities and ecosystems in their new or abandoned habitats. Thus, understanding the dynamics of species’ ranges is fundamental to understanding the dynamics of biodiversity. This is a core task of modern biology, greatly relevant to society, especially owing to ongoing global anthropogenic change that has already caused modifications of many species' ranges as well as biodiversity loss [2,3,8], and is likely to cause more change and loss in the near future [8].

The task of forecasting species’ distributions in the light of expected climate change is undoubtedly complex and challenging. Species' intrinsic properties (e.g. mating system, dispersal ability, intrinsic growth rate, niche requirements, plastic and adaptive potential for niche evolution, etc.) vary greatly, and may even vary among populations within species. This variation will influence species’ responses to a changing environment. Responses of individual species will interact, modifying communities and ecosystem processes. Unravelling these connections requires detailed theoretical and empirical knowledge of the inextricably linked roles of ecological and evolutionary processes that shape and modify species' ranges. This theme issue examines our current state of knowledge regarding the drivers of range changes and provides new hypotheses, alongside original empirical and theoretical results.

We hope that the topics addressed, and the answers delivered, in this theme issue will help to identify key factors determining long-term population persistence and shed light on how and why they vary among species. Furthermore, we hope that the theme issue will pave the way for better forecasting of species’ ranges and community composition, given a scenario of interest, including the rate at which potential range changes will occur, and their consequences. We believe that providing answers of this type is essential for designing successful policies and management actions aimed at conserving biodiversity or mitigating its loss and maintaining ecological resilience. While the theme issue consists of two parts (part I [7,9–18] and part II [19–28]), they should be viewed as a coherent unit. In what follows, we briefly introduce the topics addressed in the theme issue. In the final contribution [28], Bridle & Hoffmann provide an overview of the conclusions, recommendations and outstanding issues raised.

## 2. Single species perspectives

Most investigations of range limits and dynamics until now have taken a single-species perspective, relating distributions to environmental gradients that do not change in response to evolutionary or ecological changes in the focal species [29]. Despite great theoretical (e.g. [30–33]) and empirical (e.g. [2–6,8,34,35]) progress in understanding how processes like gene flow and ecological variation shape adaptation at range edges, many open questions remain, some of which are the focus of contributions to this theme issue.

From an ecological point of view, a species' range is expected to end where environmental conditions exceed the limits of its ecological niche, i.e. where its population growth rate at low density is no longer positive. However, this limit may not be realized owing to historical factors, such as temporal changes in the environment, stochastic variation in conditions or spatial barriers that take time to overcome. Three comparative studies address the impact of adaptive constraints on range limits and range sizes [9,10,19], while Holt and co-authors [21] explore the impacts of temporal variation in environmental conditions, which can sometimes lead to wider range limits than expected in stable environments. Niche limits may be exceeded locally, within the species’ range, as well as at its geographical limits [27]. Understanding these ecological limits is critical for the management of range change, as discussed here for non-native marine species [18].

Adding an evolutionary perspective can modify this view of range limits, turning the question to the limits of adaptation: why do populations that are close to their ecological limits not evolve ways to cope better with local conditions, thus expanding their niche and their range (e.g. [36,37])? Theoretical treatments of this question have focused on the role of gene flow and the efficacy of selection in small, marginal populations on continuous gradients or in source-sink pairs. In this issue, theory is extended to varying environmental conditions in a metapopulation [22], to the role of plasticity and the way it evolves in response to the nature of environmental variation [12], to the effect of drift, migration and demographic stochasticity on the risk of extinction in peripheral populations [14], and to the costs and benefits of dispersal in small populations [20]. Empirical studies also consider the role of plasticity at range margins [10,11,23]. Effects of dispersal may be influenced by the breeding system, which Dawson-Glass & Hargreaves [13] consider in relation to pollen limitation at range margins. If a species is able to increase its range, for example following an environmental change, additional eco-evolutionary processes may come into play. One that is considered here is the spread of underdominant mutations, which might have important consequences for the future structure of populations [15].

## 3. Community perspectives

Classical ecological theory emphasizes the key contribution of biotic interactions to establishing range limits [38]. This means that at least some of the variation in the rates and extents of species' range modifications seen under environmental change (e.g. [8]) is likely to be explained by the complex interplay of direct and indirect effects within species interaction networks [39,40]. For example, Stewart *et al*. [25] investigate the role of phenological synchrony between the range-shifting butterfly *Aricia agestis* and its novel plant hosts at its range edge. Because climate affects the phenology of host plant and butterfly independently, and may, therefore, erode novel host suitability, host shifts may be transitory. Range shifts are, therefore, likely to be dynamic and complex, as climate change impacts the spatial and temporal distributions of multiple interacting species.

Because biotic interactions regulate population dynamics and abundance, they are important agents of selection that act in concert with the abiotic environment across a species’ range. O'Brien *et al*. [24] discuss how ecological constraints imposed by antagonistic biotic interactions can reduce fitness and increase the steepness of environmental gradients, thereby sharpening limits to adaptation at range margins; alternatively, adaptation to new biotic interactions, such as host shifts [24,25], might facilitate rapid range expansion. Biotic interactions can also influence selection at range limits via trade-offs in responses to abiotic and biotic factors, as illustrated by a simple model developed by Alexander *et al*. [26]. Importantly, ecological and evolutionary ‘limits’ should not only be thought of as occurring at the edges of a species' geographical distribution. As Parmesan & Singer [27] show, species’ ranges constitute mosaics of environmental stress, driven as much by behaviour and microclimatic exposure as by macroclimatic conditions, so that species might frequently meet ‘extremes’ within the centre of their distribution. Selection can push populations to limits of adaptation within such environments, rendering (meta)populations vulnerable to climatic variability or ongoing directional climate change. Here again, biotic interactions can play a key role, because selection, for example to avoid predation or match host phenology [26,27], can help push species towards their thermal limits. Together, studies in this theme issue emphasize that the community context is essential to understanding evolutionary constraints throughout species ranges and that alterations to biotic interactions have both ecological and evolutionary consequences for the dynamics of species' ranges as environments change [25–27].

## 4. Conservation and management implications and possible actions

In addition to knowledge about the relevant evolutionary and ecological processes, understanding the situations where evolution across ranges might occur rapidly (e.g. at an expanding edge) versus those where it will be more constrained (e.g. at a trailing edge) will be valuable information for managers seeking to promote or constrain range expansions or conserve marginal populations [41]. This context might also influence the range of actions available to managers (e.g. genetic or demographic support, such as ‘evolutionary rescue’, assisted colonization, and provisioning of refuge sites) [42–44]. Such potential for rapid evolutionary responses questions the usefulness of species distribution models that lack evolutionary components in assessing species' and ecosystems’ vulnerability and management [45]. Range limit dynamics will also affect the predictability of (future) species' ranges, for example using climate envelope models, which remain an important component of risk assessments [46].

A more immediate problem for managers and conservation practitioners is how to delimit populations of species, potential routes and rates of connectivity among populations, and not least to determine the optimal design of nature reserves [47]. This is very important in spatial planning of ‘green infrastructure’ in both terrestrial and aquatic environments, to provide plots of large enough size and quality for species to thrive. Jahnke & Jonsson [16] review the literature to evaluate when biophysical approaches that model dispersal potential can be used to support seascape genetic/genomic analysis in assessing metapopulation dynamics and connectivity. Holman *et al*. [18] compare current and historical distributions of non-native ascidian species along the coast of South Africa using both genetic and inventory data. They also evaluate the usefulness of environmental DNA (eDNA) in addressing this type of question. eDNA has been proposed as a key tool in detecting non-native species at an early stage of occurrence and could contribute important data on range size, genetic composition and the early history of expansion (or contraction), which will be very useful for managing threatened native and thriving non-native species. It remains an open question whether one method alone, or a combination of several approaches, is the best way to evaluate the incidence of range shifting species when their abundance is low, for example in the early stages of introductions of non-native species, or when endangered species are rare. Finally, the role of human vectors is a much-discussed topic among managers and scientists, not least in marine ecosystems where the shipping industry, through transport and release of ballast water, risks the movement of millions of larvae of marine species [48]. The study by Hudson *et al*. [17] poses the question of whether, after a new species is established in an area, continued transport of propagules (e.g. marine larvae travelling in ballast water) will blur the historical footprints of colonization and finally homogenize the genetic structure of the species.

## 5. Concluding remarks

This theme issue provides insight into several factors involved in the dynamics of species' ranges, including spatial and temporal heterogeneity of biotic and abiotic environmental variables, and species-specific intrinsic properties. However, more work is needed to unravel potential generalizations of the patterns reported, and to make direct use of them to improve forecasting of future species' ranges in a pragmatic manner, e.g. to identify key ecological tipping points and dangerous levels of environmental change. To this end, more studies that directly link theory with empirical and experimental data, alongside more meta-analyses on factors implicated in shaping species' ranges, will be especially valuable. This will allow us to better understand the dynamics of species' ranges in the face of changing environments and, in particular, to establish a solid framework for quantifying the rate at which species' ranges are expected to change, both within and at the edge of their geographical distributions. Bridle & Hoffmann provide a thoughtful summary, alongside a number of most fruitful recommendations for future work on the topic, in the final contribution of the theme issue [28].

This theme issue was aimed to advance our understanding of the factors and mechanisms involved in limiting and/or modifying species' ranges and we hope that the many insightful contributions will inspire more studies on this most urgent topic.

## Data accessibility

This study has no additional data.
